# 0600. Altered electrical activity of the heart in pigs during apnoea

**DOI:** 10.1186/2197-425X-2-S1-P41

**Published:** 2014-09-26

**Authors:** B-A Tudor, KH Wodack, D Lebherz-Eichinger, CJ Trepte, GA Roth, DA Reuter, CG Krenn

**Affiliations:** Medical University of Vienna, Vienna, Austria; University Hospital Hamburg-Eppendorf, Hamburg, Germany

## Introduction

Cardiac arrhythmias are a major cause of morbidity and mortality. Their overall incidence is reported to vary between 14 and 19 percent in adult patients admitted to intensive care units [1]. Even in patients without prior heart pathologies, an electrical instability could be detected during repolarisation shortly before cardiac arrest with a high resolution ECG, not recordable with a conventional ECG [1]. Furthermore the effects of disease pattern and intensive care measures e.g. sepsis and elevated intracranial pressure and the administration of pressure support on heart rate and variability is poorly described and understood. In contrast even minor circadian beat to beat variations might precede the occurrence of potentially life threatening arrhythmias. Thus circadian haemodynamic profiles might thus be a marker of potentially life threatening arrhythmias and an early predictor of prognosis. It seems therefore of importance to recognize even small changes of the circadian cardiac rhythm as early as possible.

## Objectives

Changes in ventilation such as hypoxia are a major cause of haemodynamic alterations. We analysed changes of beat-to-beat cardiac activity during experimental apnoea in swine with a high resolution ECG. Obtained results may offer new insights in the development of alterations in cardiac electrical activity and thus heart lung interactions in critically ill patients.

## Methods

Four healthy pigs (36kg) were anesthetized with Sevofluran and Fentanyl according to protocol and electrodes were fixed on the prepared skin. To analyse the cardiac electric activity during normal anaesthesia and phases of three-minutes-apnoe the leads record I, II and III and reconstruct pursuant to Einthoven's equation aVR, aVL, aVF, at a 1000 Hz sampling rate, obtaining continuous ten-minute recordings (Lab System^Th^ Pro - Bard electrophysiology U.S.A.).

## Results

Results obtained from 12 apnoea-phases demonstrate that from the beginning of the apnoea the R-peak increases beat-to-beat additionally up to 0.25 mV (p< 0,05), reaching its maximum after nine seconds. This variation persisted for the entire duration of the apnoea. After restarting ventilation, the following R-peaks decreased within the next eleven seconds to their pre-value. Furthermore a reduction of the heart rate during the phase of apnoea could be observed. Other ECG data of the pigs remained unchanged during the time of apnoea.

## Conclusions

In otherwise healthy pigs, haemodynamic alterations could already be detected seconds after the onset of apnoea. With regard to comorbidities of ICU patients, it seems reasonable that changes in cardiac electric activity based on heart lung interactions in humans during their ICU stay might be observed even earlier. They furthermore might serve as early markers for the occurrence of arrhythmias and disease severity.
